# Prevalence, incidence and concomitant co-morbidities of type 2 diabetes mellitus in South Western Germany - a retrospective cohort and case control study in claims data of a large statutory health insurance

**DOI:** 10.1186/s12889-015-2188-1

**Published:** 2015-09-03

**Authors:** Michael W. J. Boehme, Gisela Buechele, Julia Frankenhauser-Mannuss, Jana Mueller, Dietlinde Lump, Bernhard O. Boehm, Dietrich Rothenbacher

**Affiliations:** State Health Office Baden-Wuerttemberg - Landesgesundheitsamt Baden-Württemberg im Regierungspräsidium Stuttgart, Nordbahnhofstrasse 135, D-70191 Stuttgart, Germany; Institute of Epidemiology and Medical Biometry, Ulm University, Helmholtzstrasse 22, D-89081 Ulm, Germany; AOK - Allgemeine Ortskrankenkasse Baden-Württemberg, Presselstraße 19, D-70191 Stuttgart, Germany; Division of Endocrinology and Diabetes, Ulm University Medical Centre, Ulm University, Albert-Einstein-Allee 23, D-89081 Ulm, Germany; LKC School of Medicine, Metabolic Disease Research Program, Nanyang Technological University Singapore and Imperial College London, 50 Nanyang Drive, Research Techno Plaza, X-Frontiers Block, Singapore, 637553 Singapore

## Abstract

**Background:**

Type 2 diabetes mellitus (T2DM) has become a world-wide epidemic. This chronic metabolic disease has a major impact on life expectancy and on quality of life. The burden of this disease includes a number of co-morbidities. However, estimates of prevalence, incidence and associated diseases as well as the current temporal development and regional differences are largely missing for South Western Germany.

**Methods:**

Lifetime diagnosis-based prevalence, incidence and presence of concomitant co-morbidities were examined between the years 2007 and 2010 in the claims data set of all insured persons of the AOK Baden-Wuerttemberg, a large statutory health insurance. The analysis was based on the respective WHO-ICD-10 codes. Data were standardized for age and sex on the residential population of about 10 million inhabitants of South Western Germany.

**Results:**

The total study cohort involved approximately 3.5 million persons each year. The standardized diagnosis-based prevalence (SDP) of T2DM rose from 6.6 %, 7.4 %, 8.0 %, up to 8.6 % in the years 2007 to 2010. Yearly SDP was between 14.0 % and 18.9 % at an age range of 60 to 64 years and between 26.7 % and 31.8 % at an age of 75 years or older. In the year 2010 the regional distributions of standardized diagnosis-based prevalence were between 7.6 % and 11.6 %, respectively. Incidence rates were 8.3 in 2008, 7.8 in 2009, and 8.7 in 2010 (all rates per 1000). The excess disease risk (odds ratio) of T2DM was for adiposity 2.8 to 3.0, hypertension 2.4 to 3.7, coronary heart disease 1.8 to 1.9, stroke 1.7 to 1.8, renal insufficiency 2.8 to 3.4, and retinopathy 2.8 to 2.9 in the years 2007 to 2010. These co-morbidities appeared several years earlier compared to the non-diabetic population.

**Conclusions:**

T2DM is common and increasing in South Western Germany. In particular a quarter of the population in higher ages was afflicted by T2DM. Interestingly a region-specific pattern was observed as well as an increase in numbers during earlier years in life. Our data underline the need for diabetes awareness programmes including early diagnosis measures as well as structured and timely health surveys for major diseases such as T2DM and its concomitant co-morbidities.

**Electronic supplementary material:**

The online version of this article (doi:10.1186/s12889-015-2188-1) contains supplementary material, which is available to authorized users.

## Background

Type 2 diabetes mellitus (T2DM) is one of the most frequent chronic metabolic diseases. According to the recent German Health Interview and Examination Survey for Adults (DEGS1 survey) in Germany 7.2 % suffer from all types of diabetes [1]. Chronic hyperglycaemia can damage many organ systems and represents a considerable health challenge and economic burden for the individual as well as for the society [2–5]. The prevalence, lifetime risk, and concomitant co-morbidities determine the burden of the disease [3, 6–8]. In particular micro- and macro-vascular complications including coronary heart diseases, myocardial infarction, stroke, retino-, neuro-, or nephropathy and arterial occlusive disease are important [6, 8–11]. These complications have a direct negative impact on the patient but also to the society in general by significantly increasing the healthcare costs [12–17]. Beside functional limitations in daily life, T2DM can lead to a shortening of the life expectancy by several years [8, 18, 19].

There is evidence that the prevalence of T2DM has risen considerably over the last decades [1, 14]. In addition, the data of the DEGS1 survey show regional differences in Germany with a trend of lower prevalences in the southern parts [1]. A similar gradient of known T2DM was found in an analysis based on the results from up to six population-based studies in Germany [20, 21]. As of today only the analysis of claims data of one other statutory health insurance has determined a single T2DM’s diagnosis-based prevalence of 4.85 % for South Western Germany [22]. However, no further analyses of the T2DM’s prevalence and their time-related development have been reported for South Western Germany, which is comprised of a relatively prosperous society with an average income in the upper third of Germany [23]. Furthermore, no study has addressed regional differences of T2DM’s prevalences within this region.

The aim of this retrospective cohort study in the state Baden-Wuerttemberg with a population of over 10 million was to investigate incidence, prevalence, and temporal trends over several years as well as regional patterns of T2DM within claims data of the members of the AOK Baden-Wuerttemberg, a large health insurance in South Western Germany. The respective data were evaluated after adjustment for age and sex and standardized on the residential population of South Western Germany. Moreover, in a supplementary analysis the excess risk of typical concomitant co-morbidities was determined.

## Methods

### Study population

The routine claims data of the AOK Baden-Wuerttemberg, a large statutory health insurance, was used for this DiMBaWue-study (diabetes mellitus in Baden-Wuerttemberg study). The AOK Baden-Wuerttemberg includes about 4 million insurants in South Western Germany. The respective state Baden-Wuerttemberg of the federal republic of Germany has over 10 million inhabitants.

### Ascertainment of prevalent diabetes mellitus

All insured persons of the AOK Baden-Württemberg permanently enrolled for at least one calendar year between 2007 until 2010 were included in this study. The cohort population included about 3.5 million persons per year (Table 1). To identify people with diabetes the recorded claims diagnoses and the prescriptions of medications were used according to the slightly modified algorithm of the investigations of claims data of the AOK Hessen [24, 25]. We identified persons with claims related to T2DM considering the diagnoses E11-E14 according to the 10th revision of the International Classification of Diseases only (ICD-10). However, diagnosis coded with E13 were excluded from the analysis due to the frequently use of this code for coding of pancreoprive diabetes, which pathophysiologically has similarities to type 1 but not to T2DM. A diabetes-related claim had to be coded at least in 3 of 4 quarters of a year. Alternatively, repeated prescriptions of anti-diabetic drugs (ATC code A10A or A10B) in more than one quarter of a year had to be documented to qualify as T2DM case. The recording of one prescription of an anti-diabetic drug in combination with a diabetes-related claim (includes measurement of glucose or HbA1c) was also valuated as indicative for T2DM. To analyze the prevalence of diabetes in different districts of South Western Germany the postal ZIP-code of the insured person was used. The data of each calendar year were separately analyzed using the described algorithm each time.

**Table 1:** Basic information of the used claims data

	2007	2008	2009	2010
Total number of insured persons	4 048 709	3 931 988	3 906 411	3 954 172
Age in years (mean)	43.0	43.5	43.7	43.7
Sex (men & women)	47.1 % & 52.9 %	47.0 % & 53.0 %	47.1 % & 52.9 %	47.2 % & 52.8 %
Number of persons insured permanently over the respective year (study population)	3 538 793	3 483 739	3 459 461	3 492 326
Proportion of the included study population compared to total number of insured persons (%)	87.4 %	88.6 %	88.6 %	88.3 %
Age in years (mean)	44.4	44.8	40.0	45.0
Sex (men & women)	46.2 % & 53.8 %	46.3 % & 53.7 %	46.4 % & 53.6 %	46.4 % & 53.6 %

### Ascertainment of incident diabetes mellitus

For identification of incident type 2 diabetes the same procedure was applied. However, only persons continuously enrolled in the health insurance plan for two consecutive calendar years were considered. For each identified person with a diabetes-related claim in the second year the previous year was checked for the presence of any diabetes-related claim. If no prior diabetes-related claim was detected, the patient was considered as incident case in the second of the two years.

### Ascertainment of concomitant co-morbidities

To identify relevant concomitant co-morbidities and to determine the burden of disease data sets of persons with and without T2DM were checked for the following ICD-10 codes: adiposity (E66), hypertension (I10-I13), coronary heart diseases (I20-I22, I24-I25), chronic renal insufficiency (N18, N08.3), stroke and cerebral circulatory disorders (I63-I64), and retinopathy (H35, H36.03). In addition, for estimating the excess risk of disease in a case–control study, each insured person with type 2 diabetes was age- and sex-matched to one control of the same calendar year. Controls were randomly chosen among the subjects without diabetes (no documented diagnosis E10-E14) and had to be permanently insured during this calendar year. All insured persons with T2DM were included in this case–control study.

### Statistical methods

The statistical analysis was performed in a two-step approach. The primary data analysis and the compilation of the cohort were carried out through the AOK Baden-Wuerttemberg (Department of Business Intelligence) by internal analysis of their claims data. The AOK Baden-Württemberg provided for further analysis an anonymized dataset with disease rates aggregated on age groups and sex for the period 2007 until 2010. The age groups comprised 5 year-categories except the first age group covering the ages 0 until 20 years.

Prevalence and incidence rates were standardized on the total population of South Western Germany considering the resident population at 31st of December of the respective year (data source: Statistical Federal Office, Wiesbaden 2013, GENESIS-Online, feature code 12411–0012 - last accessed 10.03.2013 [26]). Only the determination of the excess risk of selected co-morbidities was performed with the original data set from the case–control cohort using no further standardization. The population of the 44 distinct districts of South Western Germany was standardized on the averaged resident population of the year (data source: Statistical Offices of the Federation and the Countries, 2013, Regionalstatistik GENESIS-Online feature code 173-32-4 - last accessed 20.02.2013 [27]).

To calculate confidence intervals for the rates, the variance estimation considered the direct standardization [28]. Differences in time trends between 2007 and 2010 were quantified with the Cochran-Armitage-trend test. To compare persons with and without diabetes standardized prevalence ratios (SPR) or - in the case–control study - odds ratios were calculated and complemented with 95 % confidence intervals (CI). These calculations and preparations of all figures were performed with SAS Version 9.3 (SAS Institute Inc., Cary, NC, USA).

To assess the shift of the co-morbidity distributions for persons with T2DM the semi-maximum values of the concomitant co-morbidities were calculated. The age groups were transformed into a continuous age in years using the class means. The prevalence curves of each concomitant co-morbidity were modelled stratified for diabetes with fractional polynomials (Software r Version 2.15.1 http://cran.r-project.org, Paket mfp) [29, 30]. For each model the fit (R^2^) was assessed. The intersection of the semi-maximum values and the prevalence curves were determined with the Ridders method (Software r Version 2.15.1 http://cran.r-project.org, Paket pracma) [31]. The interpolated age at the respective intersections was subsequently compared between persons with and without type 2 diabetes by calculating the difference.

## Results

Table 1 summarizes baseline information of the claims data used. The number of total persons insured varied between 3.9 and over 4.0 million for the years 2007 to 2010. Approximately 3.5 million were permanently insured for the whole year. This reflects between 87.4 % and 88.6 % of all insured persons and between 36.4 % and 37.7 % of the total residual population of South Western Germany. The mean ages in the four years varied between 44.4 and 45.0 years and were comparable among the respective years.

### Prevalence and incidence of diabetes

**Fig. 1:**
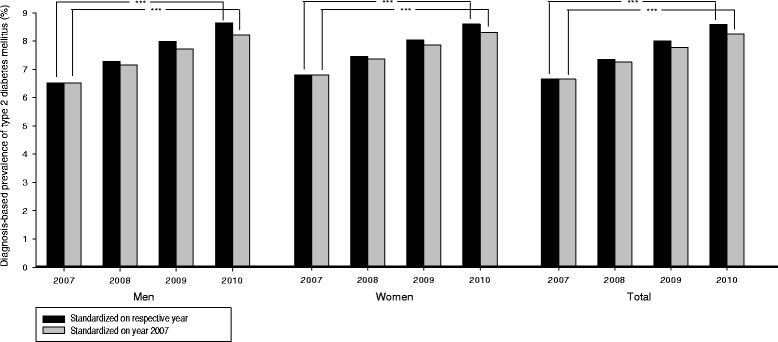
Lifetime diagnosis-based prevalence of type 2 diabetes mellitus for the years 2007 to 2010. Only persons of the AOK Baden-Wuerttemberg insured all four quarters of a year were analyzed and the results standardized for age and sex on the total residual population of South Western Germany. (***p-value of trend test <0.001)

Figure 1 shows a rise in the estimated standardized lifetime diagnosis-based prevalence of T2DM from 6.6 % (men (m): 6.5 %, women (w) 6.8 %) in the year 2007 to 7.3 % (m: 7.3 %, w: 7.4 %) in 2008 and 8.0 % (m: 8.0 %, w: 8.0 %) in 2009 up to 8.6 % (m: 8.6 %, w: 8.6 %) in the year 2010 (overall differences among the years p < 0.001, for confidence intervals see Table 2). This statistically significant increase was also evident provided that the residential population of 2007 was used as the reference for standardization.

**Table 2:** Diagnosis-based prevalence and 95 % confidence intervals (CI)

		Diagnosis-based prevalence of type 2 diabetes mellitus, standardized on the respective year			
Year		2007	2008	2009	2010
Men	Prevalence	6.5 %	7.3 %	8.0 %	8.6 %
	95 % CI	6.49-6.54	7.26-7.30	7.97-8.01	8.61-8.66
Women	Prevalence	6.8 %	7.4 %	8.0 %	8.6 %
	95 % CI	6.75-6.79	7.40-7.45	8.00-8.05	8.55-8.60
Total	Prevalence	6.6 %	7.3 %	8.0 %	8.6 %
	95 % CI	6.61-6.64	7.31-7.34	7.95-7.99	8.55-8.58

**Fig. 2:**
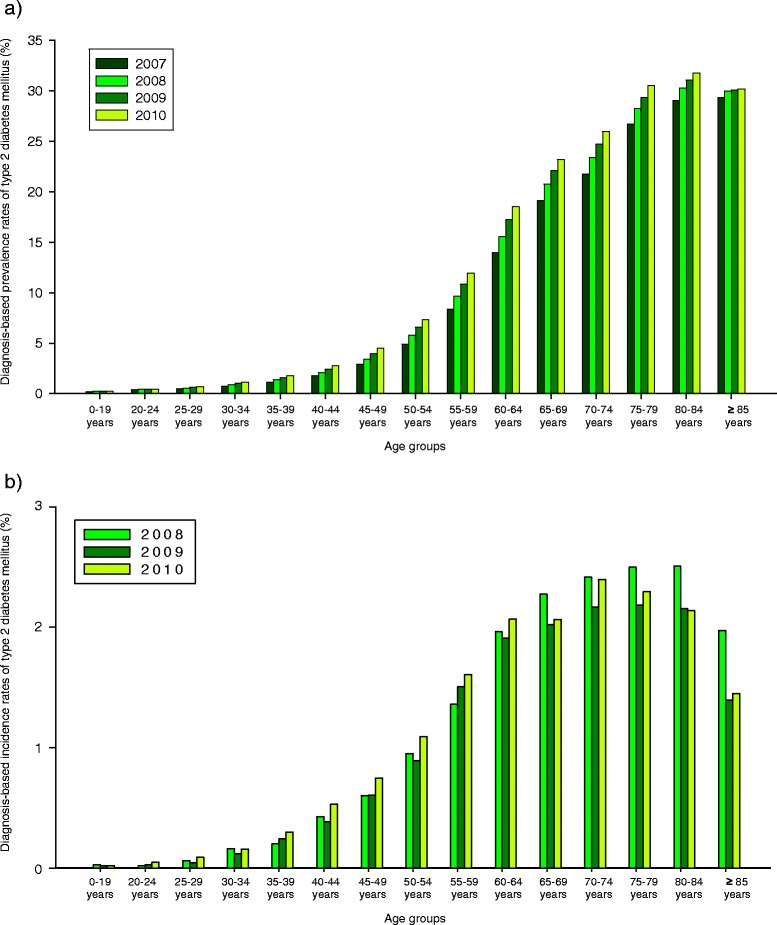
Treatment prevalence (**a**) and incidence (**b**) of type 2 diabetes mellitus of 5 years age groups. The age group specific diagnosis-based prevalence and incidence rates of insured persons of the AOK Baden-Wuerttemberg are shown for the years 2007 to 2010. Changes from 2007 to 2010 (prevalence) and 2008 to 2010 (incidence) were statistically significant different in all age groups (all *p*-values < 0.001)

Figure 2 gives the diagnosis-based prevalence (panel a) and incidence (panel b) according to 5 years age groups. An increase in the diagnosis-based prevalence was seen in all age categories already starting at younger ages. It exceeded 5 % in the age group of 50 to 54 years, was approximately 15 % in the age group of 60 to 64 years, and over 25 % in the age groups older than 75 years. With regard to the latter, the diagnosis-based prevalence remained high whereas a decrease of the respective incidences was seen. A statistically significant difference among men and women was observed. Females reached the prevalence levels in males with a time difference of about 5 years of age (data not shown in detail).

In addition, the diagnosis-based prevalence of T2DM was separately calculated for the population aged 20 years and over to avoid the influence of potential misclassification of T2DM in children and adolescent. The respective diagnosis-based prevalences were 8.3 % (m: 8.3 %, w: 8.4 %), 9.2 % (m: 9.2 %, w: 9.2 %), 9.9 % (m: 10.1 %, w: 9.9 %), and 10.6 % (m: 10.8 %, w: 10.5 %) for the years 2007 to 2010 respectively (further details see Table 3). In addition, the standardized incidence rates (all rates per 1000) of T2DM were 8.21 (m: 8.86, w 7.78) for the year 2008, 7.69 (m: 8.32, w: 7.26) for the year 2009, and 8.62 (m: 9.17, w: 8.27) for the year 2010 (further details see Table 4).

**Table 3:** Diagnosis-based prevalence and 95 % confidence intervals (CI) for the age groups 20 years and older

		Diagnosis-based prevalence of type 2 diabetes mellitus, standardized on the respective year, age group 20 years and older			
Year		2007	2008	2009	2010
Men	Prevalence	8.3 %	9.2 %	10.1 %	10.8 %
	95 % CI	8.25-8.31	9.18-9-24	10.04-10.10	10.80-10.86
Women	Prevalence	8.4 %	9.2 %	9.9 %	10.5 %
	95 % CI	8.39-8.44	9.17-9.22	9.86-9.92	10.50-10.56
Total	Prevalence	8.3	9.2 %	9.9 %	10.6 %
	95 % CI	8.31-8.35	9.15-9.19	9.91-9.95	10.61-10.65

**Table 4:** Diagnosis-based incidence and 95 % confidence intervals (CI)

		Diagnosis-based incidence of type 2 diabetes mellitus, standardized on the respective year (all rates per 1000)		
Year		2008	2009	2010
Men	Incidence (per 1000)	8.86	8.32	9.17
	95 % CI	8.78-8.94	8.24-8.40	9.09-9.26
Women	Incidence (per 1000)	7.78	7.26	8.27
	95 % CI	7.71-7.85	7.19-7.34	8.19-8.35
Total	Incidence (per 1000)	8.21	7.69	8.62
	95 % CI	8.15-8.26	7.64-7.74	8.57-8.68

An additional analysis of the data of 2010 determined the proportion of persons with T2DM on all persons with any diabetes diagnosis in the records depending on the specific ICD-10 codes used for analysis. This analysis showed considering all persons with any ICD codes for diabetes without further restrictions the following proportions for each code: 12.1 % (E10), 82.2 % (E11), 0.2 % (E12), 3.9 % (E13), and 53.0 % (E14) respectively, indicating multiple coding by the physicians. Using the respective ICD codes under the restriction that these were recorded alone and not together with other diabetes specific codes, the proportions on all persons with a diagnosis of T2DM were 40.9 % using E11, 0 % using E12 or E13, and 13.5 % using E14 only. In addition, the sole prescription of antidiabetic drugs or insulin without a connection to a diabetes specific WHO-ICD-10 code over the whole calendar year was evident in 0.1 % of all insured persons only.

### Importance of concomitant co-morbidities

**Fig. 3:**
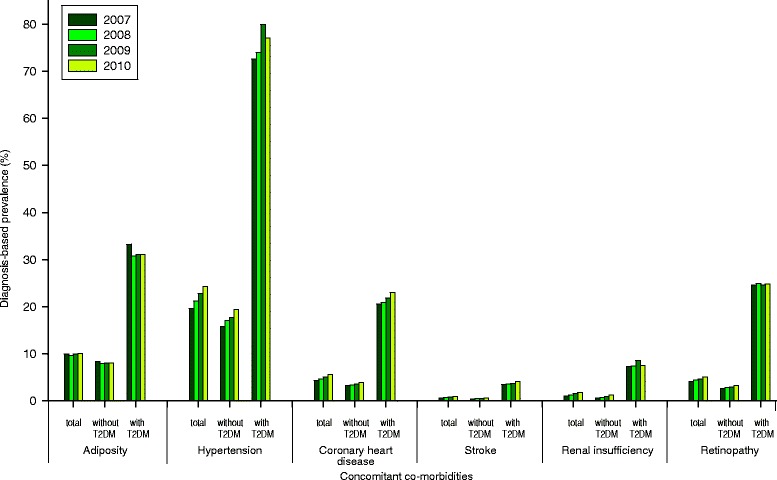
Lifetime diagnosis-based prevalence of concomitant co-morbidities for the years 2007 to 2010. The rates of insured of the AOK Baden-Wuerttemberg were stratified in persons without type 2 diabetes mellitus (T2DM) and with T2DM and standardized for age and sex on the total residual population of South Western Germany of the respective year

As displayed in Fig. 3, the comparison of T2DM associated concomitant co-morbidities showed for all examined diseases a much higher rate for persons with T2DM. For the years 2007 to 2010 the standardized diagnosis-based prevalence of persons with T2DM ranged from 30.8 % (95 % CI: 30.7-30.9) to 33.3 % (95 % CI: 33.1-33.4) for adiposity, from 72.6 % (95 % CI: 72.4-72.8) to 79.9 % (95 % CI: 79.7-80.1) for hypertension, from 20.6 % (95 % CI: 20.5-20.7) to 23.0 % (95 % CI: 22.9-23.1) for coronary heart diseases, from 3.4 % (95 % CI: 3.4-3.5) to 4.2 % (95 % CI: 4.1-4.2) for stroke, from 7.3 % (95 % CI: 7.2-7.4) to 8.6 % (95 % CI: 8.5-8.7) for renal insufficiency, and from 24.6 % (95 % CI: 24.5-24.7) to 24.9 % (95 % CI: 24.8-25.0) for retinopathy. It was considerable lower for subjects without T2DM.

Table 5 shows the corresponding standardized prevalence ratios (SPR) of the burden of concomitant co-morbidities in patients with T2DM compared to subjects without for the year 2010 after standardization to the total residual population of South Western Germany. For the data for the years 2007 to 2010 see Additional file 1. The SPR for adiposity ranged from 3.8 to 4.0, for hypertension from 4.0 to 4.6, for coronary heart diseases from 5.8 to 6.4, for stroke from 6.5 to 8.1, for renal insufficiency from 6.2 to 11.8, and for retinopathy from 7.7 to 9.5 for the respective year. However, the confidence intervals of the point estimates are wide and reveal no differences among these years for the different co-morbidities.

**Table 5:** Burden of selected concomitant co-morbidities 2010

Disease	Men			Women			Total		
(Year 2010)	Standardized prevalence rates (95 % CI)		SPR (95%CI)	Standardized prevalence rates (95 % CI)		SPR (95 % KI)	Standardized prevalence rates (95 % CI)		SPR (95 % CI)
	Without T2DM	With T2DM		Without T2DM	With T2DM		Without T2DM	With T2DM	
Adiposity	5.99 (5.97-6.01)	27.11 (26.95-27.26)	4.53 (1.87-10.96)	9.83 (9.81-9.86)	34.49 (34.32-34.66)	3.51 (1.73-7.12)	8.08 (8.07-8.10)	31.13 (31.02-31.25)	3.85 (1.78-8.35)
Hypertension	17.84 (17.80-17.87)	74.68 (74.43-74.93)	4.19 (2.50-7.02)	20.84 (20.80-20.88)	79.12 (78.87-79.38)	3.8 (2.34-6.15)	19.37 (19.34-19.40)	77.01 (76.83-77.19)	3.98 (2.42-6.54)
Coronary heart disease	4.71 (4.69-4.73)	27.77 (27.61-27.92)	5.89 (2.22-15.65)	3.32 (3.31-3.34)	18.95 (18.83-19.08)	5.70 (1.78-18.29)	3.32 (3.31-3.34)	22.99 (22.89-23.08)	5.84 (2.01-17.02)
Stroke	0.70 (0.69-0.71)	4.73 (4.67-4.80)	6.77 (0.55-83.35)	0.60 (0.59-0.61)	3.66 (3.61-3.72)	6.10 (0.4-93.4)	0.64 (0.64-0.65)	4.15 (4.11-4.19)	6.47 (0.47-89.7)
Renal insufficiency	1.43 (1.42-1.44)	8.65 (8.56-8.73)	6.06 (1.03-35.66)	1.08 (1.07-1.09)	6.69 (6.62-6.76)	6.2 (0.81-47.33)	1.23 (1.22-1.24)	7.57 (7.52-7.63)	6.17 (0.92-41.5)
Retinopathy	2.61 (2.60-2.62)	23.71 (23.57-23.85)	9.08 (2.53-32.61)	3.82 (3.80-3.84)	25.86 (25.71-26.00)	6.77 (2.31-19.82)	3.24 (3.23-3.25)	24.83 (24.73-24.93)	7.66 (2.41-24.37)

The standardized prevalence ratios are shown for adiposity and several vascular determined concomitant diseases comparing insured persons with and without type 2 diabetes mellitus (T2DM). The prevalence rates were standardized for age and sex on the residual population of South Western Germany of the respective year with 95 % confidence intervals (CI) (SPR = standardized prevalence ratio)

**Fig. 4:**
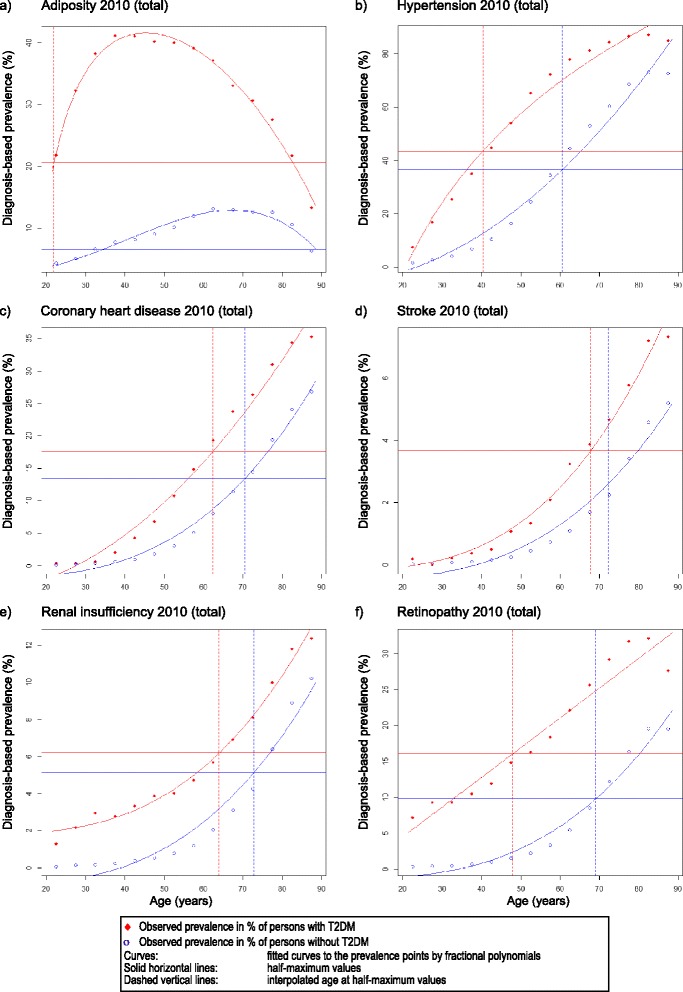
Comparison of the prevalence rates of concomitant co-morbidities. The age shifts of prevalence rates in persons with and without type 2 diabetes mellitus in the AOK Baden-Wuerttemberg determined at the semi-maximum values are shown for adiposity (**a**) and several concomitant co-morbidities (**b**-**f**) for the year 2010. The horizontal lines show the semi-maximum diagnosis-based prevalence and the vertical lines the respective age belonging to the semi-maximum values. For this analysis the age groups of 20 years and older were considered only

The further evaluation of the standardized semi-maximum values of age group specific occurrence of disease for the year 2010 revealed an age shift in case of all examined concomitant co-morbidities (Table 6). For this analysis the age groups of 20 years and older were considered only to avoid the influence of potential misclassification of T2DM in children and adolescent. The respective semi-maximum values were reached several years earlier by persons with T2DM in comparison with the persons without T2DM. The shift of the semi-maximum to earlier ages was on average 11 to 12 years for adiposity, 19 to 23 years for hypertension, 7 to 8 years for coronary heart diseases, 4 to 5 years for stroke, 8 to 12 years for renal insufficiency, and 21 to 26 years for retinopathy. For the data for the years 2007 to 2010 see Additional file 2. The goodness of fit (R^2^) was > 0.90 for most of the model adaptations indicating a very good fitting of the estimated curves. Only slight differences were seen among the years 2007 to 2010 when results were stratified by sex. Figure 4 shows a graphical comparison of the age shift of prevalence rates in persons aged 20 years and older with and without T2DM for the year 2010.

**Table 6:** Semi-maximum prevalence and interpolated age shift of selected concomitant co-morbidities 2010

Disease	Men			Women			Total		
(Year 2010)	Age at semi-maximum (prevalence at semi-maximum, R^2^)		Age shift (years)	Age at semi-maximum (prevalence at semi-maximum, R^2^)		Age shift (years)	Age atsemi-maximum (prevalence at semi-maximum, R^2^)		Age shift (years)
	Without T2DM.	With T2DM		Without T2DM	With T2DM		Without T2DM	With T2DM	
Adiposity	38.0 (5.07, 0.95)	24.7 (16.97, 0.99)	13	31.8 (7.88, 0.96)	<20* (24.50, 1.00)	NC	34.3 (6.59, 0.96)	21.9 (20.56, 0.99)	12
Hypertension	56.5 (34.74, 0.97)	37.7 (42.39, 0.98)	19	60.8 (37.58, 0.97)	40.9 (44.21, 0.97)	20	60.5 (36.56, 0.97)	40.3 (43.54, 0.98)	20
Coronary heart disease	69.7 (16.74, 0.99)	61.5 (21.51, 0.98)	8	72.8 (12.38, 0.97)	68.9 (16.53, 1.00)	4	70.5 (13.42, 0.99)	62.5 (17.64, 0.98)	8
Stroke	71.1 (3.05, 0.97)	67.6 (4.45, 0.98)	5	74.2 (2.45, 0.94)	70.2 (3.45, 0.99)	4	72.2 (2.59, 0.97)	67.8 (3.66, 0.99)	4
Renal insufficiency	73.0 (6.97, 0.96)	66.1 (8.13, 0.97)	7	74.4 (4.54, 0.93)	66.0 (5.64, 0.98)	8	72.7 (5.12, 0.96)	64.0 (6.19, 0.99)	9
Retinopathy	70.3 (9.53, 0.98)	45.7 (15.60, 0.95)	25	69.0 (10.32, 0.96)	48.9 (16.39, 0.92)	20	69.1 (9.79, 0.97)	47.9 (16.07, 0.94)	21

The age and prevalence of concomitant co-morbidities at semi-maximum values are shown comparing insured persons with and without type 2 diabetes mellitus (T2DM) after standardization for age and sex on the residual population of South Western Germany. R^2^ is the goodness of fit from the respective regression model calculating the curves. For this analysis the age groups of 20 years and older were considered only

(* The intersection with the fitted curve and the semi-maximum is located at a time point below 20 years. Therefore, the age shift is not calculable (NC).)

Finally the excess risk of disease was analyzed in the nested case–control study estimating the prevalence rates and the relative risk (odds ratio) for selected concomitant co-morbidities. The results for the year 2010 are shown in Table 7. For all included comorbidities a significantly higher odds ratio was seen for T2DM. The excess disease risk (odds ratios) for insured persons with T2DM compared to subjects without diabetes varied for adiposity from 2.8 to 3.0, for hypertension 2.4 to 3.7, coronary heart diseases 1.8 to 1.9, stroke 1.7 to 1.8, renal insufficiency 2.8 to 3.4, and retinopathy 2.8 to 2.9 for the years 2007 to 2010. For the data for the years 2007 to 2010 see Additional file 3.

**Table 7:** Excess risk of disease for selected concomitant co-morbidities 2010

Disease	Men			Women			Total		
(Year 2010)	Prevalence rates (95 % CI)		Odds Ratio (95 % CI)	Prevalence rates (95 % CI)		Odds Ratio (95 % CI)	Prevalence rates (95 % CI)		Odds Ratio (95 % CI)
	With T2DM	Controls		With T2DM	Controls		With T2DM	Controls	
Adiposity	25.98 (25.72-26.24)	10.24 (10.07-10.40)	3.08 (3.02-3.14)	31.99 (31.73-32.24)	13.64 (13.48-13.81)	2.98 (2.93-3.03)	29.31 (29.13-29.50)	12.13 (12.01-12.24)	3.00 (2.97-3.04)
Hypertension	77.40 (76.95-77.84)	57.08 (56.69-57.46)	2.58 (2.53-2.62)	81.93 (81.52-82.34)	63.14 (62.78-63.50)	2.65 (2.61-2.69)	79.91 (79.61-80.22)	60.44 (60.18-60.71)	2.60 (2.58-2.63)
Coronary heart disease	30.52 (30.24-30.80)	18.6 (18.38-18.81)	1.92 (1.89-1.96)	21.35 (21.14-21.56)	12.55 (12.39-12.71)	1.89 (1.86-1.92)	25.43 (25.26-25.60)	15.24 (15.11-15.37)	1.90 (1.87-1.92)
Stroke	5.37 (5.25-5.49)	3.23 (3.14-3.32)	1.70 (1.64-1.76)	4.16 (4.07-4.25)	2.54 (2.47-2.61)	1.67 (1.61-1.73)	4.70 (4.63-4.77)	2.85 (2.79-2.90)	1.68 (1.64-1.73)
Renal insufficiency	9.56 (9.40-9.72)	3.15 (3.06-3.24)	3.25 (3.14-3.36)	7.38 (7.25-7.5)	2.18 (2.11-2.25)	3.57 (3.45-3.70)	8.35 (8.25-8.45)	2.61 (2.56-2.67)	3.39 (3.31-3.48)
Retinopathy	25.27 (25.02-25.53)	9.14 (8.99-9.29)	3.36 (3.29-3.43)	27.38 (27.14-27.61)	13.17 (13.01-13.34)	2.48 (2.44-2.53)	26.44 (26.27-26.61)	11.38 (11.27-11.49)	2.80 (2.76-2.84)

The prevalences of concomitant co-morbidities are shown comparing all persons with type 2 diabetes mellitus (T2DM) and respective controls in a case–control-study. The controls are matched individually by age and sex. The prevalence rates as well as the respective odds ratios are calculated with 95 % confidence intervals (CI). This analysis was performed without standardization

### Regional patterns of diagnosis-based prevalence in the distinct districts of South Western Germany

**Fig. 5:**
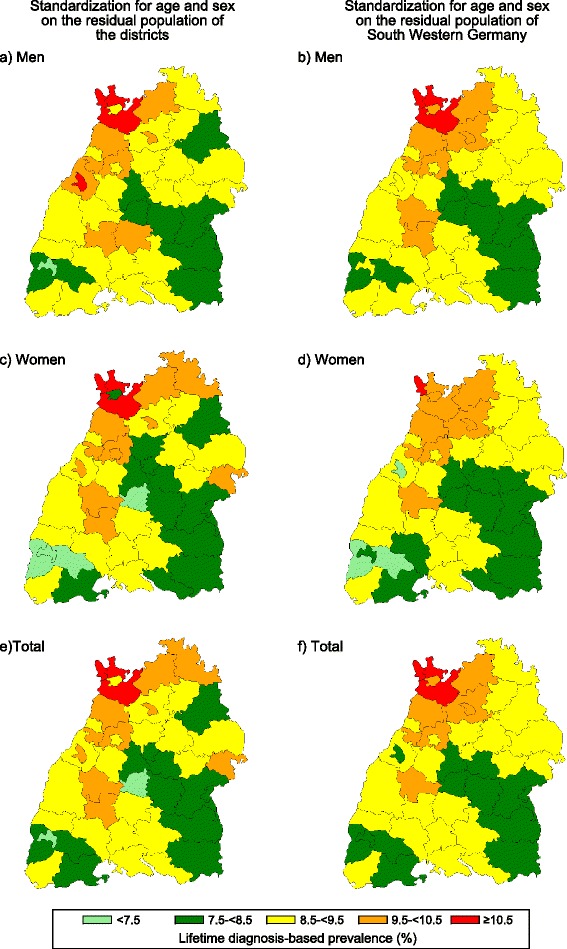
Regional lifetime diagnosis-based prevalence. The regional variation in the diagnosis-based prevalence of type 2 diabetes mellitus is shown for the year 2010 after standardization on the residual population of the respective distinct districts (**a**, **c**, **e**) or on South Western Germany (**b**, **d**, **f**)

As displayed in Fig. 5 considerable differences were seen in the lifetime diagnosis-based prevalence of T2DM in the 44 distinct districts of South Western Germany in the year 2010. After standardization on the residual population of the districts, for determination of the district-related burden of disease and e.g. direct regional assessments of health care needs, the diagnosis-based prevalences of T2DM ranged between 7.2 % (95 % CI: 7.1-7.3) and 11.5 % (95 % CI: 11.4-11.6) (m: 7.4 % (95 % CI: 7.3-7.6) to 11.5 % (95 % CI: 11.3-11.6); w: 7.0 % (95 % CI: 6.8-7.1) to 11.5 % (95 % CI: 11.3-11.7)). After standardization of the data on the residential population of South Western Germany (Fig. 5 b, d, and f), enabling a direct comparison of the distinct districts irrespective of age and sex distribution, diagnosis-based prevalences were between 7.6 % (95 % CI: 7.5-7.6) and up to 11.6 % (95 % CI: 11.58-11.62) (m: 7.9 % (95 % CI: 7.84-7.89) up to 11.6 % (95 % CI: 11.56-11.62); w: 7.2 % (95 % CI: 7.23-7.27) up to 11.6 % (95 % CI: 11.59-11.64). Non evident pattern between the prevalence and urban or rural characteristics of the districts was found. The diagnosis-based prevalence of several districts changed to a higher as well as to a lower prevalence class after standardization of the data on the residual population of South Western Germany (Fig. 5).

## Discussion

We provide estimates of prevalence, incidence and concomitant co-morbidities of T2DM in a population from South Western Germany, which is comprised of over 10 million inhabitants. Our results are based on claims data of the AOK Baden-Wuerttemberg, a large statutory health insurance including about 4 million insurants in South Western Germany. The data show a clear increase of the lifetime diagnosis-based prevalence of T2DM from the year 2007 to 2010, which is still seen after excluding the demographic changes during this time period. Notably, in older people more than 25 % suffered from T2DM, associated with considerable comorbidity. Given the current demographic transition, the burden of T2DM and of diabetes associated co-morbidities will pose an increasing challenge to the health care system. Our data underline the need for early screening programmes as well as early treatment of co-morbidities to encounter this dramatic development. However, several limitations has to be considered concerning the analysis of claims data like non-uniform or incorrect coding, incomplete diagnosis of the physicians, lack of standardized criteria of diagnosis, missing validity checks of the diagnosis, and structural differences of the membership of health insurances.

### Lifetime diagnosis-based prevalence of T2DM

The prevalence data of our study are comparable with recent investigations of health insured persons of the AOK Hessen, Germany, a comparable statutory health insurance. Those studies reported a diagnosis-based prevalence of 9.7 % for all types of diabetes together after standardization on the population of Germany in 2009 [14, 25]. Furthermore, the recent nationwide representative DEGS1 survey found a slightly lower prevalence of known diabetes of 7.2 % in Germany in the year 2011. However in this survey the diagnosis of diabetes was established by self-report of diabetes or self-statement of antidiabetic drugs only, which can lead to underestimation. Interestingly, a special analysis of the data of the DEGS1 survey revealed a marked influence of the membership to different health insurances on the prevalence ranging for diabetes altogether from 3.8 % up to 9.0 % with the highest value for the health insurance we investigated in the present study [1]. Taking into account that about 5 to 10 % of all patients with diabetes are considered to have type 1 diabetes the prevalences of our study revealed the same magnitude as estimated in the DEGS1 survey for the AOK health insurance data or the analysis of the data of the AOK Hessen.

A considerably lower diagnosis-based prevalence of T2DM was found in the recent analysis of another statutory health insurance in Germany. This analysis showed a prevalence of T2DM of only 4.7 % for Germany and 4.8 % for South Western Germany by cumulative evaluation of the insured persons of the years 2008 to 2010. However, this analysis is only partly comparable with our results as it cumulated the data of three years. In addition, the structure of the membership was different to our population, which might be in correspondence with the results of the DEGS1 survey the main reason for the different prevalences. Furthermore, their algorithm used for analysis considered the WHO-ICD-10 code E11 for the detection of persons with T2DM only. This excluded the substantial amount of insured persons with T2DM coded as E14 alone, which applies in our investigation about an eighth of all patients with a diabetes diagnosis [22]. In contrast, we used an established algorithm first applied in insured persons of the AOK Hessen with a slight modification to focus on T2DM only [24, 25].

In addition to the prevalence of known diabetes a considerable amount of undiagnosed diabetes may exist in the population. The prevalence of undiagnosed diabetes was estimated at 2.1 % in the DEGS1 survey for the age group of 18 to 79 years [32]. Furthermore, the KORA survey 2000 in Southern Germany revealed for the age group of 55 to 74 years a prevalence of undiagnosed diabetes of 8.2 % after screening with an oral glucose tolerance test. This prevalence was nearly as high as the prevalence of known diabetes with 8.7 % [33]. This is of particular interest due to the considerable amount of already existing co-morbidities like polyneuropathy, increased mortality and the increased requirement of drugs of persons with undiagnosed diabetes [17, 34–36]. Therefore, the considerable amount of undetected diabetes might have led to an underestimation of the age group related prevalence of T2DM as well as of the prevalences of concomitant co-morbidities in our study.

The incidence rates of T2DM with 7.8 to 8.7/1000 in our study were about twice as high as those determined with 3.7/1000 person-years for South Western Germany in a recent analysis of the claims data of another health insurance. However, the prevalence of T2DM was about half as well in that investigation, which may explain the differences of the incidences found [22]. A higher incidence rate of self-reported T2DM with 9.3/1000 person-years was recently shown for the South of Germany by the analysis of five regional population-based studies of the years 1997 to 2010. However, those studies investigated participants aged 45 years and over, a group with increased risk of developing a T2DM and the data are therefore not directly comparable with our study [21]. The incidence rates found in our study are in the same range with those in other comparable countries. An incidence rate of 7.3/1000 person-years was determined for T2DM in England. Studies in the USA and Canada found incidence rates of diabetes of 8/1000 in 2008 (USA) and 8.2/1000 in 2003 (Canada) respectively [37–39].

Altogether, the prevalence of T2DM has risen considerably in Germany over the last decades. It was about 2 % in the year 1968 in Munich and 0.7 % in 1961 according to the former German Democratic Republic (GDR) diabetes register with an increase up to 4.0 % in the year 1987 [40, 41]. The German National Health Interview and Examination Survey 1997/98 found a prevalence of known diabetes of 4.3 % for men and 3.8 % for women in the total population of Germany [42]. Participants of this survey took also part in the DEGS1 survey. Comparing the results of both surveys the DEGS1 survey confirmed an increased prevalence of diabetes in Germany from 4.3 % to 7.2 % in about 10 years [1]. Estimates of a sample of insured persons of the AOK Hessen have also shown an increasing diagnosis-based prevalence of diabetes in Germany over the years from 7.2 % in 1998 up to almost 10 % in recent years [14, 24, 25]. In our study a significant increase of the prevalence of T2DM over the four years was still found after standardization of all data on the residential population of 2007 suggesting a demographic independent factor. Beside a real increase of the occurrence of T2DM in the last years an additional increase could partially be due to a rising awareness by the medical professions as well as by the population in general.

In addition, our study provides evidence of an increase of the prevalence of T2DM already starting at early adolescence and in relatively young age groups. This is of special interest due to a possible higher rate of complications in early-onset T2DM [43]. The increase and the temporal course of the diagnosis-based prevalences of T2DM are comparable to current studies in the area of Germany and the steep increase with advancing age was found in other investigations also [1, 24, 25]. In the retirement age over a quarter of the population suffered T2DM. In former studies indications were found for a decrease of prevalence in the age groups over 80 or 90 years, a pattern which was not seen in our study [44]. However, we could find a decrease of the incidence of T2DM in this age group. The constant high prevalences with advancing age despite decreasing incidence may point to a possible improvement of medical care of these patients. In addition, as the occurrence of T2DM starts to increase earlier in life, these results underline the relevance of modification of established lifestyle factors in early age already to positively influence the development of T2DM in future [45–48].

The prevalence of diabetes in our study may considerably differ from results estimated from claims data of other health insurance companies for various reasons. Altogether, insured persons of the AOK are known to show a relatively high prevalence of T2DM while insured persons of private health insurances show a lower one as recently determined in the DEGS1 survey. This observation fact is present despite standardization for age and sex. There are disparities in the structure of the membership of insured persons with respect to socio-demographic and life-style factors, which might be one explanation [1, 14, 24, 49]. These underlying differences could partly be overcome in further investigations by the use of pooled data from different health insurances.

Several studies have shown differences in the prevalence and incidence of diabetes in different regions of Germany. A tendency towards a lower prevalence can be found in the South of Germany [1, 22, 20, 21]. In addition, the investigation of distinct districts in our study showed a considerable range of the diagnosis-based prevalence of T2DM within South Western Germany. The differences were still evident after standardization for age and sex. These different regional prevalence patterns could be due to differences in the awareness and frequency of the diagnosis of T2DM, the non-uniform use of the WHO-ICD-10 codes by physicians in different regions, or regional differences in additional factors like social and economic status, regional deprivation, intensive traffic burden or different genetic disposition for T2DM as revealed e.g. in the DEGS1 survey or as pooled analysis of up to six population-based studies [1, 20, 21, 50, 51]. Unfortunately, we had no suitable data to explore these differences further.

In the present study insurants with T2DM were determined according to the established algorithm of the analysis of the claims data of the AOK Hessen. This algorithm had successfully been used in these studies over years [24, 25]. In order to analyze T2DM only the algorithm was slightly modified by excluding the code E10, which codes type 1 diabetes. Furthermore the code E13 was excluded as well due to the frequently use of this code for coding of pancreoprive diabetes. This form of diabetes is characterized by direct destruction of the insulin producing cells due to several reasons (e.g. toxic) and not by altered insulin-sensitivity and production. Therefore, pancreoprive diabetes shows pathophysiological similarities to type 1 diabetes. In addition, our additional analysis of the use of the different ICD-10 codes for diabetes by the physicians revealed considerable multiple coding by the physicians and showed that it is useful to consider more than one code. E.g. the code E14 concerning undefined diabetes was used alone for coding without other diabetic specific codes in 13.5 %. Most of these patients are usually regarded as T2DM and would have been missed out in the analysis by exclusion of E14 in the analysis. This may be one reason for the lower prevalence of T2DM found in the analysis of the claims data of another health insurance [22].

### Concomitant co-morbidities

The case control analysis of the present study shows dramatic excess risks for concomitant co-morbidities due to vascular alterations as well as adiposity for patients with T2DM. The respective excess risks were between twofold and threefold on average. These differences were even more pronounced when comparing patients with T2DM with the total population of South Western Germany. The excess risks found are comparable with results of other studies [6, 8, 52, 53].

Concerning these concomitant co-morbidities it is of further particular importance, that most of them can be modified by therapeutic interventions. E.g. the occurrence of concomitant co-morbidities by T2DM is associated with increasing fasting blood glucose and can be reduced by a good adjustment of blood glucose levels [6, 8, 9, 54]. Furthermore, a sufficient therapy of hypertension can markedly reduce the mortality risk related to vascular complications of diabetes. A reduction of the systolic blood pressure by 10 mm Hg can reduce the mortality of ischemic heart diseases by approximately 30 % and of strokes by 40 % [55–57]. The increased risk of persons with adiposity to develop a T2DM and their about three-fold increased mortality should be an important reason for preventive activities. Teenagers with adiposity have an up to 8-fold increased risk to develop a T2DM [58–61]. Furthermore, adiposity increases the risk of stroke as well as increased blood sugar and duration of diabetes [62–64]. In addition, approximately a third of all patients with T2DM have a renal insufficiency 10 years after diagnosis with a high risk to develop end-stage renal disease and diabetic retinopathy [65–68]. The risk of blindness is considerably increased for persons with T2DM. In current examinations it is about 2 ½-folds higher for T2DM than in the general population [68–71]. Altogether, the occurrence of concomitant co-morbidities of patients with diabetes has declined in the last decades, but the burden of disease remained nearly unchanged due to the increased prevalence of diabetes [72].

In this context it is of particular interest, that the concomitant co-morbidities in patients with T2DM do not only appear far more frequently but also far earlier in life compared to persons without T2DM. These factors contribute both to an increased burden of disease, and to a reduced average life expectancy of patients with T2DM [3, 6–8, 18, 19]. Moreover, the treatment of concomitant co-morbidities cause on average three-quarters of all medical costs of patients with T2DM at present [73].

Concerning the general population the determined prevalences of concomitant co-morbidities in our analysis were tendentiously lower than in some other studies. This was especially the case for adiposity and to a minor extent for coronary heart disease, stroke, and chronic renal insufficiency. The lifetime prevalence in the general population of Germany has been described in current surveys between 20 to 23 % for adiposity, at about 32 % for hypertension and between 7 to 9.3 % for coronary heart disease [74–79]. These differences can be due to the use of claims data of a health insurance company in our study whereby only physician recorded diagnoses can be investigated missing the undiscovered ones in the general population. In contrast, systematic screening approaches in study populations can also discover asymptomatic disease. The use of routine claims data can lead to a considerable underestimation especially for diseases with an asymptomatic period, which can delay clinical manifestation and diagnosis. In addition, the ICD-coding of physicians for their claims can considerably differ depending on the presented complaints of their patients and on the extent of considering individual diagnoses. Furthermore, the ICD-coding in administrative data has only a limited sensitivity for identifying persons with diabetes [80]. Finally, the social status of the insured persons can influence the prevalence of morbidities in the population as well. E.g. this was found for low social status and increase of hypertension or diabetes [1, 79].

### Strength and limitations

We analyzed the claims data of the total number of all insured persons of the AOK Baden-Wuerttemberg, a large statutory health insurance in South Western Germany. Thereby about two-fifth of the total population of South Western Germany was included. The completed claims data of the period from 2007 until 2010 were included in the analysis. The established algorithm for claims data of the analysis of insurants of the AOK Hessen was used for analysis with the slight modification of using the WHO-ICD-10 codes E11, E12 and E14 only. The relatively recent data and the large number should ensure a good external validity of our data.

Limitations of a study based on claims data should be considered. These are e.g. the non-uniform or incorrect use of the WHO-ICD-10 codes by the physicians, structural differences between the membership of the particular health insurances, the completeness of recording of diseases by the physicians, and the lack of detection of undiagnosed T2DM. Additional restrictions are the lack of the use of standardized criteria of diagnosis of diseases and the missing comparability and validity check of the physician’s diagnoses. All these limitations may have influenced the analysis and may have led to an under- or overestimation of disease as well as to an age shift towards later years in the present estimation.

A minor restriction of the present data analysis consists in the use of the complete data set of insured persons of the AOK Baden-Wuerttemberg for South Western Germany. These data contain a small proportion of persons with residence in Germany but residing outside of the State of Baden-Wurttemberg. It was assumed in this context that no essential alteration of the finally calculated prevalence arose from including these additional insured persons especially by residency in the border zone of South Western Germany.

## Conclusions

In summary our survey shows that T2DM is common in South Western Germany and affects more than a quarter of the population in the higher age groups. It is also associated with considerable putatively treatable co-morbidities, which are present more frequently at earlier ages. T2DM showed major regional differences and occurred more often earlier in life. Our data underline the need for structured and timely health surveys, in view of the diabetic epidemics early diagnosis as well as prevention programmes for major diseases such as diabetes mellitus and its concomitant treatable co-morbidities.

## Additional files

Additional file 1:
**Burden of selected concomitant co-morbidities (2007–2010).** The table shows the standardized prevalence ratios for adiposity and several vascular determined concomitant diseases comparing insured persons with and without type 2 diabetes mellitus (T2DM) for the years 2007 to 2010. The prevalence rates were standardized for age and sex on the residual population of South Western Germany of the respective year with 95 % confidence intervals (CI) (SPR = standardized prevalence ratio) (Boehme et al. Additional file 1). (PDF 111 kb) Additional file 2:
**Semi-maximum prevalence and interpolated age shift of selected concomitant co-morbidities (2007–2010).** The Table shows the age and prevalence of concomitant co-morbidities at semi-maximum values comparing insured persons with and without type 2 diabetes mellitus (T2DM) after standardization for age and sex on the residual population of South Western Germany of the respective year. The data for the years 2007 to 2010 are shown. R^2^ is the goodness of fit from the respective regression model calculating the curves. For this analysis the age groups of 20 years and older were considered only (Boehme et al. Additional file 2). (PDF 112 kb) Additional file 3:
**Excess risk of disease for selected concomitant co-morbidities (2007–2010).** The table shows the prevalences of concomitant co-morbidities comparing all persons with type 2 diabetes mellitus (T2DM) and respective controls in a case–control-study for the years 2007 to 2010. The controls are matched individually by age and sex. The prevalence rates as well as the respective odds ratios are calculated with 95 % confidence intervals (CI). This analysis was performed without standardization (Boehme et al. Additional file 3). (PDF 110 kb)

## Abbreviations

AOK: Allgemeine Ortskrankenkasse, a general local statutory medical insurance
ATC: Anatomical Therapeutic Chemical classification system
CI: Confidence interval
DEGS1: German Health Interview and Examination Survey for Adults
ICD: International Classification of Disease
RKI: Robert Koch Institute
SPR: Standardized prevalence ratio
SDP: Standardized diagnosis-based prevalence
T2DM: Type 2 diabetes mellitus
WHO: World Health Organization
