# Supplementary material for: Prevalence, incidence and concomitant co-morbidities of type 2 diabetes mellitus in South Western Germany - a retrospective cohort and case control study in claims data of a large statutory health insurance
Source: BMC Public Health. 2015 Sep 3;15:855. doi: 10.1186/s12889-015-2188-1 (PMC4559219; doi:10.1186/s12889-015-2188-1)
Supplement: Additional file 1: — Burden of selected concomitant co-morbidities (2007–2010). The table shows the standardized prevalence ratios for adiposity and several vascular determined concomitant diseases comparing insured persons with and without type 2 diabetes mellitus (T2DM) for the years 2007 to 2010. The prevalence rates were standardized for age and sex on the residual population of South Western Germany of the respective year with 95 % confidence intervals (CI) (SPR = standardized prevalence ratio) (Boehme et al. Additional file 1). (PDF 111 kb) [file 12889_2015_2188_MOESM1_ESM.pdf]

Additional file 1: **Burden of selected concomitant co-morbidities (2007-2010).**
The standardized prevalence ratios are shown for adiposity and several vascular determined concomitant diseases comparing insured persons with and without type 2 diabetes mellitus (T2DM). The prevalence rates were standardized for age and sex on the residual population of South Western Germany of the respective year with 95% confidence intervals (CI) (SPR = standardized prevalence ratio)

Disease	Year	Men			Women			Total		
		Standardized prevalence rates (95% CI)		SPR (95%CI)	Standardized prevalence rates (95% CI)		SPR (95%KI)	Standardized prevalence rates (95% CI)		SPR (95%CI)
		Without T2DM.	With T2DM		Without T2DM.	With T2DM		Without T2DM.	With T2DM	
Adiposity	2007	6.01 (5.99-6.03)	28.67 (28.49-28.85)	4.77 (1.98-11.5)	10.35 (10.32-10.38)	37.19 (37.00-37.39)	3.59 (1.8-7.16)	8.32 (8.31-8.34)	33.26 (33.13-33.39)	4 (1.87-8.54)
	2008	5.78 (5.75-5.80)	26.40 (26.24-26.56)	4.57 (1.86-11.25)	9.77 (9.74-9.79)	34.47 (34.29-34.65)	3.53 (1.73-7.18)	7.92 (7.91-7.94)	30.78 (30.66-30.91)	3.88 (1.78-8.48)
	2009	5.93 (5.90-5.95)	26.80 (26.65-26.96)	4.52 (1.86-11.01)	9.93 (9.90-9.95)	34.61 (34.44-34.79)	3.49 (1.72-7.06)	8.09 (8.07-8.11)	31.07 (30.95-31.19)	3.84 (1.77-8.32)
	2010	5.99 (5.97-6.01)	27.11 (26.95-27.26)	4.53 (1.87-10.96)	9.83 (9.81-9.86)	34.49 (34.32-34.66)	3.51 (1.73-7.12)	8.08 (8.07-8.10)	31.13 (31.02-31.25)	3.85 (1.78-8.35)
Hypertension	2007	14.17 (14.14-14.21)	69.52 (69.25-69.80)	4.91 (2.77-8.68)	17.40 (17.37-17.44)	75.39 (75.11-75.67)	4.33 (2.57-7.3)	15.81 (15.79-15.84)	72.60 (72.40-72.80)	4.59 (2.66-7.91)
	2008	15.46 (15.43-15.50)	71.28 (71.01-71.55)	4.61 (2.66-7.99)	18.63 (18.60-18.67)	76.41 (76.14-76.68)	4.10 (2.47-6.8)	17.08 (17.06-17.11)	73.98 (73.79-74.17)	4.33 (2.56-7.33)
	2009	16.02 (15.99-16.06)	80.01 (79.74-80.28)	4.99 (2.92-8.54)	19.31 (19.27-19.35)	79.83 (79.56-80.09)	4.13 (2.52-6.80)	17.71 (17.68-17.74)	79.89 (79.70-80.08)	4.51 (2.70-7.55)
	2010	17.84 (17.80-17.87)	74.68 (74.43-74.93)	4.19 (2.50-7.02)	20.84 (20.80-20.88)	79.12 (78.87-79.38)	3.8 (2.34-6.15)	19.37 (19.34-19.40)	77.01 (76.83-77.19)	3.98 (2.42-6.54)
Coronary heart disease	2007	3.73 (3.71-3.74)	24.40 (24.23-24.56)	6.55 (2.20-19.48)	2.80 (2.79-2.82)	17.19 (17.05-17.32)	6.13 (1.73-21.65)	3.23 (3.22-3.24)	20.57 (20.46-20.68)	6.36 (1.97-20.55)
	2008	3.97 (3.96-3.99)	25.21 (25.05-25.37)	6.34 (2.20-18.27)	2.89 (2.88-2.91)	17.26 (17.13-17.39)	5.97 (1.72-20.72)	3.38 (3.37-3.39)	20.94 (20.84-21.04)	6.19 (1.96-19.53)
	2009	4.30 (4.28-4.32)	26.45 (26.29-26.60)	6.15 (2.22-17.04)	3.06 (3.04-3.07)	17.86 (17.73-17.98)	5.84 (1.74-19.65)	3.61 (3.60-3.62)	21.80 (21.70-21.90)	6.04 (1.98-18.4)
	2010	4.71 (4.69-4.73)	27.77 (27.61-27.92)	5.89 (2.22-15.65)	3.32 (3.31-3.34)	18.95 (18.83-19.08)	5.70 (1.78-18.29)	3.94 (3.92-3.95)	22.99 (22.89-23.08)	5.84 (2.01-17.02)
Stroke	2007	0.45 (0.44-0.46)	3.83 (3.77-3.90)	8.50 (0.39-186.16)	0.40 (0.40-0.41)	3.07 (3.01-3.13)	7.64 (0.29-204.84)	0.42 (0.42-0.43)	3.43 (3.39-3.48)	8.09 (0.33-196.13)
	2008	0.53 (0.52-0.53)	4.10 (4.04-4.16)	7.77 (0.44-136.58)	0.46 (0.45-0.46)	3.16 (3.11-3.22)	6.93 (0.31-154.14)	0.49 (0.48-0.49)	3.60 (3.56-3.64)	7.37 (0.37-146.57)
	2009	0.59 (0.59-0.60)	4.31 (4.25-4.37)	7.24 (0.48-108.95)	0.52 (0.51-0.52)	3.26 (3.20-3.31)	6.31 (0.33-118.87)	0.55 (0.54-0.55)	3.74 (3.70-3.78)	6.80 (0.40-115.58)
	2010	0.70 (0.69-0.71)	4.73 (4.67-4.80)	6.77 (0.55-83.35)	0.60 (0.59-0.61)	3.66 (3.61-3.72)	6.10 (0.4-93.4)	0.64 (0.64-0.65)	4.15 (4.11-4.19)	6.47 (0.47-89.7)
Renal insufficiency	2007	0.74 (0.73-0.75)	8.44 (8.34-8.53)	11.41 (1.06-122.87)	0.52 (0.51-0.52)	6.27 (6.18-6.35)	12.12 (0.71-206.48)	0.62 (0.62-0.63)	7.29 (7.23-7.36)	11.73 (0.88-156.11)
	2008	0.89 (0.88-0.90)	8.62 (8.52-8.71)	9.70 (1.09-86.13)	0.63 (0.62-0.63)	6.38 (6.30-6.46)	10.18 (0.76-136.35)	0.75 (0.74-0.75)	7.42 (7.36-7.48)	9.94 (0.92-107.49)
	2009	1.09 (1.08-1.10)	9.79 (9.70-9.89)	9.01 (1.24-65.38)	0.78 (0.77-0.79)	7.58 (7.49-7.66)	9.70 (0.94-99.61)	0.92 (0.91-0.92)	8.59 (8.53-8.65)	9.37 (1.09-80.81)
	2010	1.43 (1.42-1.44)	8.65 (8.56-8.73)	6.06 (1.03-35.66)	1.08 (1.07-1.09)	6.69 (6.62-6.76)	6.20 (0.81-47.33)	1.23 (1.22-1.24)	7.57 (7.52-7.63)	6.17 (0.92-41.5)
Retinopathy	2007	2.03 (2.01-2.04)	23.22 (23.05-23.38)	11.46 (2.73-48.17)	3.15 (3.14-3.17)	25.96 (25.79-26.12)	8.23 (2.56-26.51)	2.60 (2.59-2.61)	24.65 (24.54-24.77)	9.47 (2.64-33.95)
	2008	2.20 (2.19-2.21)	23.57 (23.42-23.72)	10.71 (2.69-42.66)	3.37 (3.35-3.39)	26.08 (25.92-26.24)	7.74 (2.49-24.06)	2.81 (2.80-2.82)	24.89 (24.78-25.00)	8.87 (2.58-30.48)
	2009	2.32 (2.31-2.33)	23.46 (23.32-23.61)	10.11 (2.62-38.95)	3.45 (3.44-3.47)	25.69 (25.54-25.84)	7.44 (2.42-22.87)	2.91 (2.90-2.92)	24.63 (24.53-24.74)	8.46 (2.51-28.52)
	2010	2.61 (2.60-2.62)	23.71 (23.57-23.85)	9.08 (2.53-32.61)	3.82 (3.80-3.84)	25.86 (25.71-26.00)	6.77 (2.31-19.82)	3.24 (3.23-3.25)	24.83 (24.73-24.93)	7.66 (2.41-24.37)
