# Supplementary material for: Prevalence, incidence and concomitant co-morbidities of type 2 diabetes mellitus in South Western Germany - a retrospective cohort and case control study in claims data of a large statutory health insurance
Source: BMC Public Health. 2015 Sep 3;15:855. doi: 10.1186/s12889-015-2188-1 (PMC4559219; doi:10.1186/s12889-015-2188-1)
Supplement: Additional file 2: — Semi-maximum prevalence and interpolated age shift of selected concomitant co-morbidities (2007–2010). The Table shows the age and prevalence of concomitant co-morbidities at semi-maximum values comparing insured persons with and without type 2 diabetes mellitus (T2DM) after standardization for age and sex on the residual population of South Western Germany of the respective year. The data for the years 2007 to 2010 are shown. R2 is the goodness of fit from the respective regression model calculating the curves. For this analysis the age groups of 20 years and older were considered only (Boehme et al. Additional file 2). (PDF 112 kb) [file 12889_2015_2188_MOESM2_ESM.pdf]

Additional file 2: **Semi-maximum prevalence and interpolated age shift of selected concomitant co-morbidities (2007-2010).**

The age and prevalence of concomitant co-morbidities at semi-maximum values are shown comparing insured persons with and without type 2 diabetes mellitus (T2DM) after standardization for age and sex on the residual population of South Western Germany of the respective year. R^2 is the goodness of fit from the respective regression model calculating the curves. For this analysis the age groups of 20 years and older were considered only. (* The intersection with the fitted curve and the semi-maximum is located at a time point below 20 years. Therefore, the age shift is not calculable (NC).)

Disease	Year	Men			Women			Total		
		Age at semi-maximum (prevalence at semi-maximum, R^2)		Age shift (years)	Age at semi-maximum (prevalence at semi-maximum, R^2)		Age shift (years)	Age atsemi-maximum (prevalence at semi-maximum, R^2)		age shift (years)
		Without T2DM.	With T2DM		Without T2DM	With T2DM		Without T2DM	With T2DM	
Adiposity	2007	40.3 (5.66, 0.91)	29.1 (17.41, 0.95)	11	34.3 (8.77, 0.94)	24.0 (25.69, 0.98)	10	36.5 (7.29, 0.93)	24.7 (21.07, 0.99)	12
	2008	39.9 (5.27, 0.93)	27.1 (16.19, 0.94)	13	33.3 (8.06, 0.95)	23.6 (24.35, 0.99)	10	35.5 (6.65, 0.95)	24.2 (19.81, 0.97)	11
	2009	38.7 (5.13, 0.94)	26.6 (16.30, 0.97)	12	32.3 (8.05, 0.96)	22.3 (24.56, 0.99)	10	34.7 (6.65, 0.95)	24.0 (20.55, 0.97)	11
	2010	38.0 (5.07, 0.95)	24.7 (16.97, 0.99)	13	31.8 (7.88, 0.96)	<20* (24.50, 1.00)	NC	34.3 (6.59, 0.96)	21.9 (20.56, 0.99)	12
Hypertension	2007	61.5 (31.78, 0.98)	42.5 (40.39, 0.97)	19	62.3 (35.27, 0.97)	43.8 (42.82, 0.96)	18	62.0 (33.95, 0.97)	43.4 (41.87, 0.97)	19
	2008	60.9 (32.87, 0.98)	41.3 (40.90, 0.98)	20	61.7 (36.15, 0.97)	42.8 (43.33, 0.96)	19	61.4 (34.93, 0.97)	42.3 (42.40, 0.97)	19
	2009	61.0 (33.35, 0.98)	38.6 (43.16, 0.89)	22	61.9 (37.60, 0.97)	39.1 (42.86, 0.87)	23	61.6 (36.04, 0.98)	38.8 (42.91, 0.88)	23
	2010	56.5 (34.74, 0.97)	37.7 (42.39, 0.98)	19	60.8 (37.58, 0.97)	40.9 (44.21, 0.97)	20	60.5 (36.56, 0.97)	40.3 (43.54, 0.98)	20
Coronary heart disease	2007	71.1 (15.77, 0.99)	63.9 (20.15, 0.99)	7	74.3 (11.92, 0.95)	70.8 (15.83, 0.99)	3	72.0 (12.89, 0.98)	65.0 (16.83, 0.99)	7
	2008	70.8 (16.14, 0.99)	62.7 (20.01, 0.98)	8	73.9 (11.97, 0.96)	70.3 (15.75, 1.00)	4	71.6 (12.99, 0.98)	64.1 (16.72, 0.99)	8
	2009	70.2 (16.35, 0.99)	61.9 (20.57, 0.98)	8	73.4 (12.05, 0.96)	69.7 (16.03, 1.00)	4	71.0 (13.09, 0.99)	63.2 (17.05, 0.99)	8
	2010	69.7 (16.74, 0.99)	61.5 (21.51, 0.98)	8	72.8 (12.38, 0.97)	68.9 (16.53, 1.00)	4	70.5 (13.42, 0.99)	62.5 (17.64, 0.98)	8
Stroke	2007	73.4 (2.56, 0.96)	70.0 (3.98, 0.99)	3	76.0 (1.93, 0.91)	72.7 (3.14, 0.98)	3	74.1 (2.09, 0.95)	70.4 (3.33, 1.00)	4
	2008	72.5 (2.70, 0.96)	69.8 (4.23, 0.99)	3	75.4 (2.06, 0.93)	71.7 (3.14, 0.99)	4	73.4 (2.22, 0.96)	69.6 (3.39, 0.99)	4
	2009	72.3 (2.92, 0.97)	67.6 (3.98, 0.98)	5	74.6 (2.19, 0.94)	70.8 (3.15, 0.99)	4	72.8 (2.37, 0.96)	68.2 (3.34, 0.99)	5
	2010	71.1 (3.05, 0.97)	67.6 (4.45, 0.98)	5	74.2 (2.45, 0.94)	70.2 (3.45, 0.99)	4	72.2 (2.59, 0.97)	67.8 (3.66, 0.99)	4
Renal insufficiency	2007	75.9 (4.64, 0.92)	67.8 (8.18, 0.97)	8	76.7 (2.47, 0.90)	63.1 (5.44, 0.89)	14	74.9 (3.01, 0.94)	63.1 (6.08, 0.97)	12
	2008	75.1 (5.18, 0.93)	67.7 (8.51, 0.98)	7	76.2 (2.89, 0.91)	68.4 (5.67, 0.97)	8	74.3 (3.46, 0.94)	65.9 (6.32, 0.98)	8
	2009	74.5 (5.91, 0.95)	67.0 (9.48, 0.98)	8	75.4 (3.46, 0.92)	67.3 (6.59, 0.97)	8	73.8 (4.06, 0.95)	65.2 (7.24, 0.98)	9
	2010	73.0 (6.97, 0.96)	66.1 (8.13, 0.97)	7	74.4 (4.54, 0.93)	66.0 (5.64, 0.98)	8	72.7 (5.12, 0.96)	64.0 (6.19, 0.99)	9
Retinopathy	2007	72.4 (9.08, 0.96)	47.3 (15.15, 0.96)	25	69.9 (9.44, 0.96)	48.2 (16.41, 0.93)	22	71.0 (9.35, 0.97)	48.1 (15.92, 0.95)	23
	2008	72.2 (9.57, 0.97)	42.9 (15.06, 0.92)	29	69.6 (9.78, 0.96)	48.3 (16.43, 0.94)	21	70.6 (9.69, 0.97)	46.7 (15.91, 0.94)	24
	2009	71.4 (9.49, 0.97)	46.7 (15.08, 0.96)	25	69.5 (9.89, 0.96)	48.7 (16.22, 0.94)	21	70.0(9.62, 0.97)	48.2 (15.79, 0.95)	22
	2010	70.3 (9.53, 0.98)	45.7 (15.60, 0.95)	25	69.0 (10.32, 0.96)	48.9 (16.39, 0.92)	20	69.1(9.79, 0.97)	47.9 (16.07, 0.94)	21
