# Supplementary material for: Prevalence, incidence and concomitant co-morbidities of type 2 diabetes mellitus in South Western Germany - a retrospective cohort and case control study in claims data of a large statutory health insurance
Source: BMC Public Health. 2015 Sep 3;15:855. doi: 10.1186/s12889-015-2188-1 (PMC4559219; doi:10.1186/s12889-015-2188-1)
Supplement: Additional file 3: — Excess risk of disease for selected concomitant co-morbidities (2007–2010). The table shows the prevalences of concomitant co-morbidities comparing all persons with type 2 diabetes mellitus (T2DM) and respective controls in a case–control-study for the years 2007 to 2010. The controls are matched individually by age and sex. The prevalence rates as well as the respective odds ratios are calculated with 95 % confidence intervals (CI). This analysis was performed without standardization (Boehme et al. Additional file 3). (PDF 110 kb) [file 12889_2015_2188_MOESM3_ESM.pdf]

Additional file 3: **Excess risk of disease for selected concomitant co-morbidities (2007-2010).** The prevalences of concomitant co-morbidities are shown comparing all persons with type 2 diabetes mellitus (T2DM) and respective controls in a case-control-study. The controls are matched individually by age and sex. The prevalence rates as well as the respective odds ratios are calculated with 95% confidence intervals (CI). This analysis was performed without standardization.

Disease	Year	Men			Women			Total		
		Prevalence rates (95% CI)		Odds Ratio (95% CI)	Prevalence rates (95% CI)		Odds Ratio (95% CI)	Prevalence rates (95% CI)		Odds Ratio (95% CI)
		With T2DM	Controls		With T2DM	Controls		With T2DM	Controls	
**Adiposity**	**2007**	26.98 (26.71-27.26)	11.09 (10.92-11.27)	2.96 (2.90-3.02)	33.18 (32.91-33.45)	14.97 (14.79-15.15)	2.82 (2.78-2.87)	30.48 (30.28-30.67)	13.28 (13.15-13.40)	2.86 (2.83-2.90)
	**2008**	24.94 (24.68-25.20)	10.26 (10.10-10.43)	2.90 (2.84-2.97)	31.09 (30.83-31.35)	13.80 (13.63-13.97)	2.82 (2.77-2.87)	28.40 (28.21-28.58)	12.25 (12.13-12.37)	2.84 (2.80-2.88)
	**2009**	25.47 (25.20-25.73)	10.28 (10.12-10.45)	2.98 (2.92-3.04)	31.58 (31.32-31.83)	13.73 (13.56-13.90)	2.90 (2.85-2.95)	28.89 (28.71-29.07)	12.21 (12.09-12.33)	2.92 (2.88-2.96)
	**2010**	25.98 (25.72-26.24)	10.24 (10.07-10.40)	3.08 (3.02-3.14)	31.99 (31.73-32.24)	13.64 (13.48-13.81)	2.98 (2.93-3.03)	29.31 (29.13-29.50)	12.13 (12.01-12.24)	3.00 (2.97-3.04)
**Hypertension**	**2007**	73.14 (72.69-73.59)	53.46 (53.07-53.84)	2.37 (2.33-2.41)	79.02 (78.6-79.43)	61.38 (61.01-61.74)	2.37 (2.33-2.41)	76.45 (76.15-76.76)	57.92 (57.66-58.19)	2.36 (2.33-2.38)
	**2008**	74.60 (74.15-75.05)	54.82 (54.44-55.21)	2.42 (2.38-2.46)	79.88 (79.47-80.29)	62.24 (61.88-62.61)	2.41 (2.37-2.44)	77.57 (77.26-77.87)	59.00 (58.73-59.26)	2.40 (2.38-2.43)
	**2009**	85.29 (84.81-85.76)	56.14 (55.75-56.53)	4.53 (4.45-4.61)	84.10 (83.68-84.52)	62.81 (62.45-63.17)	3.13 (3.08-3.18)	84.62 (84.31-84.94)	59.88 (59.61-60.14)	3.69 (3.64-3.73)
	**2010**	77.40 (76.95-77.84)	57.08 (56.69-57.46)	2.58 (2.53-2.62)	81.93 (81.52-82.34)	63.14 (62.78-63.50)	2.65 (2.61-2.69)	79.91 (79.61-80.22)	60.44 (60.18-60.71)	2.60 (2.58-2.63)
**Coronary heart disease**	**2007**	27.93 (27.65-28.21)	17.36 (17.14-17.58)	1.84 (1.81-1.88)	20.67 (20.46-20.88)	12.57 (12.40-12.73)	1.81 (1.78-1.85)	23.84 (23.67-24.01)	14.66 (14.53-14.79)	1.82 (1.80-1.85)
	**2008**	28.49 (28.21-28.77)	17.67 (17.45-17.89)	1.86 (1.82-1.89)	20.38 (20.17-20.59)	12.41 (12.25-12.57)	1.81 (1.77-1.84)	23.93 (23.76-24.10)	14.71 (14.58-14.84)	1.82 (1.80-1.85)
	**2009**	29.54 (29.26-29.82)	18.02 (17.80-18.24)	1.91 (1.87-1.94)	20.65 (20.44-20.86)	12.22 (12.06-12.38)	1.87 (1.84-1.90)	24.56 (24.39-24.73)	14.77 (14.64-14.90)	1.88 (1.86-1.90)
	**2010**	30.52 (30.24-30.80)	18.6 (18.38-18.81)	1.92 (1.89-1.96)	21.35 (21.14-21.56)	12.55 (12.39-12.71)	1.89 (1.86-1.92)	25.43 (25.26-25.60)	15.24 (15.11-15.37)	1.90 (1.87-1.92)
**Stroke**	**2007**	4.62 (4.51-4.74)	2.64 (2.56-2.73)	1.79 (1.71-1.86)	3.76 (3.67-3.85)	2.15 (2.08-2.22)	1.78 (1.71-1.85)	4.14 (4.07-4.21)	2.36 (2.31-2.42)	1.78 (1.73-1.83)
	**2008**	4.85 (4.74-4.97)	2.77 (2.69-2.86)	1.79 (1.72-1.86)	3.78 (3.69-3.87)	2.26 (2.19-2.33)	1.70 (1.64-1.77)	4.25 (4.18-4.32)	2.48 (2.43-2.54)	1.74 (1.69-1.79)
	**2009**	4.97 (4.85-5.08)	2.97 (2.89-3.06)	1.71 (1.64-1.77)	3.81 (3.73-3.90)	2.40 (2.33-2.47)	1.61 (1.55-1.68)	4.32 (4.25-4.39)	2.65 (2.60-2.71)	1.66 (1.61-1.70)
	**2010**	5.37 (5.25-5.49)	3.23 (3.14-3.32)	1.70 (1.64-1.76)	4.16 (4.07-4.25)	2.54 (2.47-2.61)	1.67 (1.61-1.73)	4.70 (4.63-4.77)	2.85 (2.79-2.90)	1.68 (1.64-1.73)
**Renal insufficiency**	**2007**	9.76 (9.59-9.92)	3.61 (3.51-3.71)	2.89 (2.79-2.99)	7.26 (7.14-7.39)	2.41 (2.34-2.48)	3.17 (3.06-3.29)	8.35 (8.25-8.45)	2.93 (2.87-2.99)	3.02 (2.95-3.09)
	**2008**	9.94 (9.77-10.1)	4.02 (3.92-4.12)	2.63 (2.55-2.72)	7.37 (7.25-7.50)	2.64 (2.56-2.71)	2.94 (2.84-3.04)	8.50 (8.40-8.60)	3.24 (3.18-3.30)	2.77 (2.71-2.84)
	**2009**	11.08 (10.91-11.26)	4.22 (4.12,4.33)	2.83 (2.74-2.92)	8.56 (8.43-8.70)	2.95 (2.87-3.03)	3.08 (2.99-3.18)	9.67 (9.56-9.78)	3.51 (3.44-3.57)	2.94 (2.88-3.01)
	**2010**	9.56 (9.40-9.72)	3.15 (3.06-3.24)	3.25 (3.14-3.36)	7.38 (7.25-7.5)	2.18 (2.11-2.25)	3.57 (3.45-3.70)	8.35 (8.25-8.45)	2.61 (2.56-2.67)	3.39 (3.31-3.48)
**Retinopathy**	**2007**	25.16 (24.90-25.43)	8.88 (8.72-9.03)	3.45 (3.38-3.53)	27.66 (27.42-27.91)	13.03 (12.86-13.20)	2.55 (2.51-2.60)	26.57 (26.39-26.75)	11.22 (11.1-11.33)	2.86 (2.83-2.90)
	**2008**	25.38 (25.12-25.64)	9.01 (8.85-9.17)	3.43 (3.36-3.51)	27.72 (27.47-27.96)	13.21 (13.05-13.38)	2.52 (2.48-2.56)	26.69 (26.51-26.87)	11.37 (11.26-11.49)	2.84 (2.80-2.88)
	**2009**	25.23 (24.97-25.49)	8.97 (8.82-9.13)	3.42 (3.35-3.50)	27.28 (27.04-27.52)	12.84 (12.68-13.01)	2.55 (2.50-2.59)	26.38 (26.20-26.56)	11.14 (11.03-11.25)	2.86 (2.82-2.90)
	**2010**	25.27 (25.02-25.53)	9.14 (8.99-9.29)	3.36 (3.29-3.43)	27.38 (27.14-27.61)	13.17 (13.01-13.34)	2.48 (2.44-2.53)	26.44 (26.27-26.61)	11.38 (11.27-11.49)	2.80 (2.76-2.84)
